# Older persons’ thoughts about death and dying and their experiences of care in end-of-life: a qualitative study

**DOI:** 10.1186/s12912-020-00514-x

**Published:** 2020-12-16

**Authors:** Johanna Tjernberg, Christina Bökberg

**Affiliations:** Department of Health Sciences, Faculty of Medicine, Lund University, P.O. Box 157, SE-221 00 Lund, Sweden

**Keywords:** Older persons, Nursing homes, Palliative care, Death, Dying, End-of-life care

## Abstract

**Background:**

Few studies have focused on how older persons living in nursing homes perceive their last period of life. Furthermore, previous research on older persons’ perceptions of death and dying is limited. Hence, there is an urgent need to explore their experiences during their final period in life.

**Aim:**

To explore thoughts about death and dying and experiences of care in end-of-life among older persons living in nursing homes.

**Methods:**

This study employed a qualitative approach including individual interviews with 36 older persons living in Swedish nursing homes. Questions related to quality of life; physical health; thoughts about death, dying, and the future; and experiences related to the living condition and environment were asked. The interview transcripts were analysed through content analysis. The study was approved by the Regional Ethics Review Board (reference number: 2015/4).

**Results:**

The analysis resulted in the identification of three main thematic categories: *The unavoidable and unknown end of life*, *Thoughts on control* and *Living your last period of life at a nursing home*. The older persons did not fear death itself but had some worries about dying. Spending the last stage of life at a nursing home contributed to different thoughts and feelings among the older persons. With a few exceptions, older persons characterized life at the nursing home as boring and felt they were surrounded by people who did not belong there.

**Conclusions:**

This study indicates a need for older persons to talk about death, dying and end-life issues. Furthermore, this study highlighted that the co-residence of cognitively healthy persons and persons with dementia in the same ward adversely affected cognitively healthy persons. This situation resulted in there being not enough time to both handle the care needs of persons with dementia and have the conversations that cognitively healthy persons desired, such as conversations about thoughts about existence, that could have improved their quality of life.

**Trial registration:**

NCT02708498 Date of registration 16 February 2016.

## Background

The proportion of older people in the world are about 11% and are rapidly increasing [1]. In Europe, the age group 65 years and above represents about 15% of the population [2, 3]. A large number of these older people are admitted to nursing homes, as they near end of life. In many countries up to one-third of them dies in these care settings [3]. In Sweden the majority (71%) of all people who died in 2018 were *≥* 75 years [4], and although 38% of all deaths in Sweden occur in nursing homes [5], few studies have focused on how older persons living in nursing homes perceive their last period of life [6, 7]. Furthermore, previous research concerning older persons’ perceptions of death and dying is limited [8–10]. This lack of research could be related to researchers’ desire to protect older persons, but it could also be due to a lack of interest in older persons’ last period of life. Other explanations could be a lack of knowledge among staff [10, 11] or an unwillingness and/or fear to talk to older persons about death and dying [7, 12].

In Sweden, the mean length of stay after moving into a nursing home is 730 days [13] but is decreasing [14]. Of all older persons moving into a nursing home, almost one-third die within 6 weeks [15]. Older persons are more likely to die from chronic diseases, such as heart and circulatory diseases, cancer, and dementia [4, 15], which involve complex care needs [2, 16]. It is difficult to determine when an older person’s end-of-life stage begins [17] since diseases usually proceed during many years. Early signs preceding death could be difficult for nursing home staff to detect [18], since the final year of life is often related with symptoms such as pain, depression, confusion, and distress [15, 19]. However, it can be concluded that most persons living in nursing homes are in the end of life and benefit from palliative care.

Knowledge and understanding of older persons’ perspectives about death, dying and the last period of life are prerequisites for staff working at nursing homes to enable good palliative care. Although previous research has found that older persons desire to talk about death and dying [7, 8, 20], other researchers have found that older persons living in nursing homes do not have the opportunity to discuss questions related to the last period in life [11, 15, 21]. Therefore, older persons living in nursing homes with extensive palliative care needs tend to receive care that is not adapted to their specific needs, which results in unnecessary suffering [15, 22]. Hence, there is an urgent need to explore the experiences of older persons living in nursing homes during their final period in life.

## Aim

The aim of the study was to explore thoughts about death, dying and experiences of care in end-of-life among older persons living in nursing homes.

## Methods

### Design and setting of the study

This study used a qualitative approach including interviews with 36 older persons living in Swedish nursing homes. The study was part of the project Knowledge-based Palliative Care [in Swedish, KUnskapsbaserad PAlliativ vård"], which is abbreviated as the KUPA project. For the KUPA project, a palliative intervention was implemented in 20 nursing homes. One of the goals of the project was to improve palliative care for older persons in end of life [23]. Trial registration: NCT02708498. The study was guided by COREQ guidelines.

### Context

In Sweden there are approximately 1700 nursing homes. These are accommodations provide care around the clock and are mainly for older persons (*≥* 65 years. Access to an apartment is based upon the older person’s needs and assessed by a social worker in the municipality. As a result of the ageing society and the ageing in place principal, more frail older persons receive care and service in their own home. Thus, moving into a nursing home usually occurs when the older person is too sick or frail to be able to continue living in their previous home with home care services. In 2018, approximately 88,000 older persons lived in nursing homes, most of them (67%) are women. The median age to move into a nursing home is 86.2 years for women and 83.7 years for men. Nurse assistants are the most commonly staff at nursing homes, but other professions are represented, such as registered nurses, physiotherapists, and occupational therapists [13, 24].

### Sample

The inclusion criteria were age 65 years or older, ability to communicate in Swedish and sufficient energy to participate in a one-hour interview. The exclusion criterion was cognitive impairment. At each of the nursing homes affiliated with the KUPA project, a contact person was assigned. This contact person informed older persons who fulfilled the inclusion criterion about the project and the aim of the study. They asked older persons about their interest in participating in an interview. If the older person was interested, the contact person contacted the researchers and shared the older person’s contact information. The researcher then booked a time for the interview at the nursing home with the older person. Further information about the project, voluntary participation, and the right to withdraw without any reason or consequences was provided. Time for questions was provided before the older person signed the informed consent form. In total, 36 older persons were included in this study; they had a mean age of 88 years (67–102), and most of them (20, 56%) were women. Regarding marital status, 18 (50%) of the older persons were widowed, 12 (33%) were married, four (11%) were divorced and two (6%) were single.

### Data collection

Four registered nurses with experience in meeting and talking to older persons performed the individual interviews. The nurses followed an interview guide to ask questions related to quality of life; physical health; thoughts about death, dying, and the future; and experiences related to their living conditions and environment [23]. During the interviews, the older person shared his or her spontaneous thoughts about death, dying and his or her experiences of care at the nursing home. The interviews lasted between 30 and 90 min and were tape-recorded and transcribed verbatim.

### Analyses of the transcribed interviews

The transcribed interview text was analysed through content analysis as described by Graneheim and Lundman [25]. The analysis process started with the authors (JT, CB) independently reading the whole interviews repeatedly. Then, the authors met and discussed the content. Expressions related to the study aim were identified and divided into meaning units and then condensed at a descriptive level. The condensed meaning units were then labelled with codes. The authors discussed the condensed meaning units as well as the codes. Codes were compared regarding their similarities and differences before the categories were created. Then, the authors separately read and critically reviewed the categories and sub-categories in relation to the interview text. Several combined meetings were held to reflect on the analysis process to uncover as many qualities as possible before consensus on the results was reached. Table 1 shows an example of the analysis process.

**Table 1:** Example of the analysis process

Meaning unit	Condensed meaning unit	Code	Sub-category	Category
Then, some of those living here are not healthy and should not live at a nursing home (…) she has yelled at me, and that upsets me. She yells at everybody; that’s the worst.	Some at the nursing home are not healthy and shouldn’t be there. Have been yelled at by another resident, take it personally and feel it’s the worst.	Frustration about the living situation	Living together with others	Living your last period of life at a nursing home

## Results

The analysis resulted indicated three main categories: *The unavoidable and unknown end of life*, *Thoughts on control* and *Living your last period of life at a nursing home*. In the main category *The unavoidable and unknown end of life*, which is divided into three sub-categories, describes the older persons’ worries and wishes related to the end of life as well as their approaches to death and remaining life. The second category, *Thoughts on control*, portrays the older persons different views about whether death should be controlled. The third category, *Living your last period of life at a nursing home,* describes the older persons’ thoughts, feelings and attitudes on spending the end of life at a nursing home. The three main categories and their corresponding sub-categories are presented in Table 2.

**Table 2:**
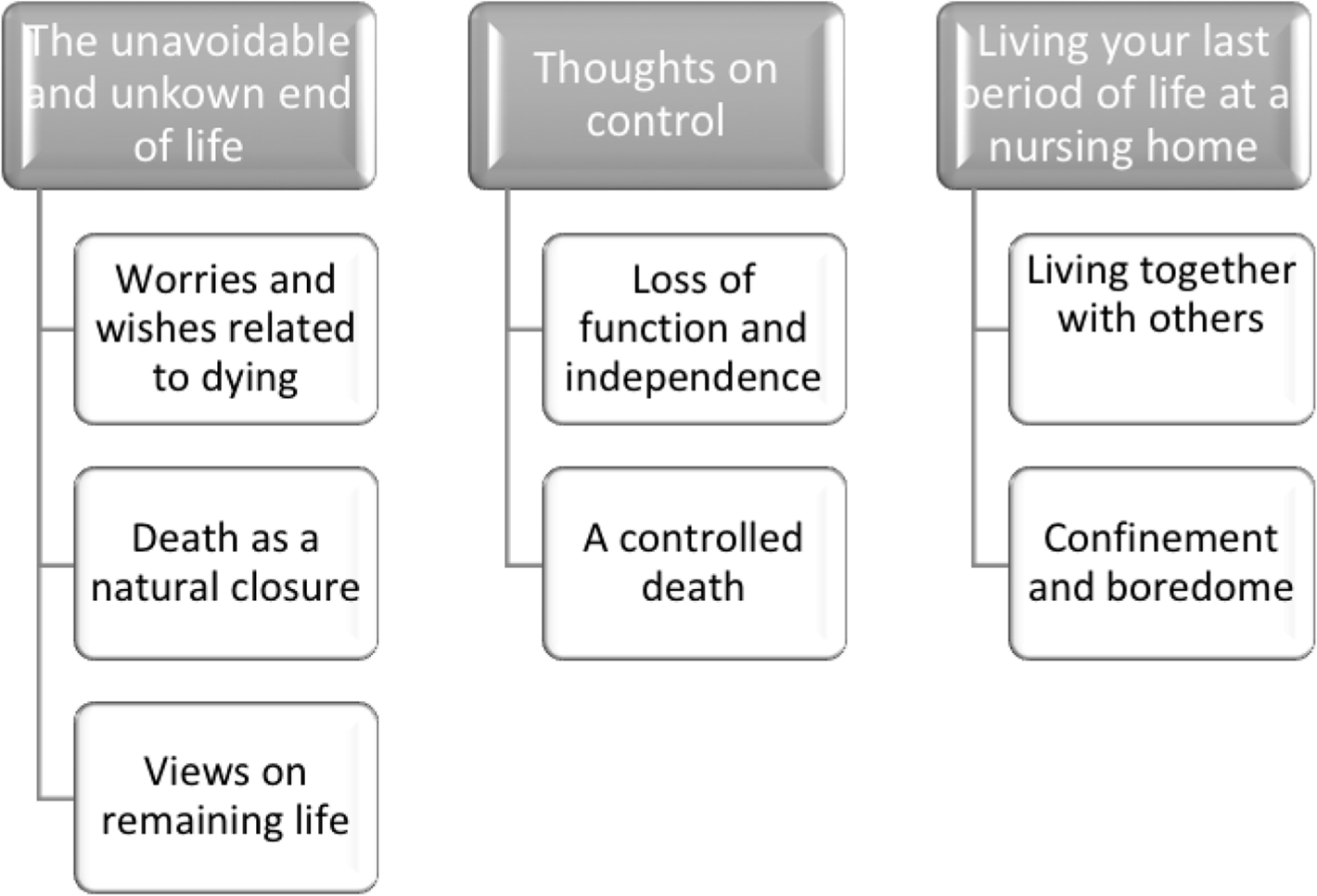
Structure of the categories and sub-categories

### The unavoidable and unknown end of life

The older persons had different perspectives on dying, death and the end of life. The older persons expressed various wishes and worries related to dying, which are described in the sub-category *Worries and wishes related to dying*. Death was perceived as something natural and an inevitable part of life, and the older persons did not particularly worry about death; however, the reasons behind their lack of worries varied, as illustrated in the sub-category *Death as a natural closure*. The older persons found it difficult to plan for the future and connected an unknown future to finding oneself in the end-phase of life – their views on finding meaning and happiness in this phase of life are described in the sub-category *Views on remaining life*.

### Worries and wishes related to dying

The older persons expressed worries related to dying. Undergoing a long and protracted death, as well as facing an end of life characterized by illness and slowly languishing away, evoked worries among the older persons. Another worry that the older persons noted concerned being in pain. The thought of dying in pain generated concerns. Some of the older persons were convinced they would receive adequate pain relief, while others worried that they would not receive the help they would need in time.

*“Possibly, you are afraid of not getting the help you need (...) because if you are lying there in pain, that is hard for both yourself and your loved ones.” – 75-80 years old*

Several of the older persons shared a wish to remain healthy until the very end of their lives. Dying quickly or suddenly was a common way that the older persons described how they would like to die. The older persons also gave examples of what would settle their worries in the face of death and help them feel peace. Those who were scared of dying in pain noted that the nursing home and caregivers gave them a sense of security. Thus, for some of the older persons, the nursing home can be viewed as a comforting place of safety and security.

*“As long as you die in a regular way (…) of course you don’t want to die in dismay” - 75–80 years old*

### Death as a natural closure

Dying evoked worries, but death itself was not feared by the older persons. Death was explained as an unavoidable part of life and therefore nothing worth being concerned about. The older persons viewed death as a natural end of life. However, the reasons why the older persons did not worry about death varied. Some had experienced near-death experiences, which resulted in them not fearing death. Others compared themselves with younger, healthy persons and argued that the death of older persons is not depressing compared to the death of younger people. Some of the older persons even portrayed death as a relief from life and thus something to long for.

*“… if you have become so weak and obsolete, then it is nice to get away from it …” – 75-80 years old*

The older persons had varied thoughts about what would happen after death. Some wished they had a faith to be comforted by, and some hoped for life after death and the ability to rest near and dear family and friends. Some of the older persons shared a view of death was a large empty room, an unknown situation they knew nothing about.

*“How afraid am I of dying. You don’t know anything. It is entirely foreign; completely empty you do not know anything” -*

*80-85 years old*

### Views on remaining life

The older persons expressed awareness of being at the end of life. Some expressed a lack of prospects or a future to look forward to, while others showed disinterest in future events. They portrayed the future as something unknown and uncontrollable. However, the older persons did not express specific worries about facing this unknown future. Not having to think about the future was described by the older persons as part of knowing they were drawing closer to the end of life.

*“I have accomplished a lot in life … so I say it is enough now.” (laughter) –85-90 years old*

The older persons described varied feelings of happiness and meaningfulness related to the remaining part of their lives. Some described difficulties finding meaning and happiness in what remained of life. These older persons viewed their lives as being over and expressed a preparedness to die.

*“I feel like I am done living, I have lived my life” -80-85 years old*

Others voiced satisfaction with themselves and what they had accomplished in life. Some of the older persons felt that their children and grandchildren brought meaningfulness to their lives. One man explained that his wife needed him due to financial reasons that gave him a sense of meaningfulness in life. A few of the older persons had a more hesitant approach, describing life as being burdensome but, at the same time, indicating an unwillingness to give up on life. Others described a feeling of being unsatisfied with themselves and their accomplishments and looked back at life with a feeling of failure.

*“Our children visit us often, we are grateful for that, having them close by” -*90-95 years old

### Thoughts of control

The older persons associated the ageing process with different kinds of losses; these losses and their impacts on the older persons are described in the sub-category *Loss of function and independence*. Death was considered uncontrollable, but the older persons also had different thoughts on how they would like to be able to control the moment of death; these thoughts are illustrated in the sub-category *A controlled death*.

### Loss of function and independence

Different types of losses connected to the ageing process were described by older persons. The loss of functions was reported as a feeling of not being able to control one’s daily activities or schedule, which was followed by a feeling of loss of independence and control. The loss of control caused a feeling of frustration, but the older persons also perceived this loss of control as a natural part of the ageing process that everyone goes through.

The older persons described decreases in physical function and the inability to perform activities they once been able to do. One man contrasted his previous ability to engage in many activities when he was healthy with his current substantial loss of physical function. He described the loss of his physical function as a transformation he had to learn to become accustomed to; however, the feeling of not being able to take care of himself caused frustration.

*“Before, I was able to do almost anything, and now I feel like a piece of shit (…) as an example, I am not able to hammer a nail into the wall; I cannot hold the nail with my left hand. So, it is a lot, but I have learnt to accept it. I just rather need to ask someone to help me.” – 75-80 years old*

Decreased physical function contributed to a feeling of lost independence among the older persons. Some described decreased control of the bladder and decreased mobility. One woman described the difficulty of getting up from a chair and noted that pain was a contributing factor. The inability take care of herself or have control of her day-to-day activities was very frustrating, and constantly being dependent on others gave her a sense of hopelessness.

*“… I have difficulties calling (…) I want to take care of myself.” –95-100 years old*

Their thoughts about losing control of physical function were accompanied by a sense of fear of being a burden. The older persons described themselves as being a burden to caregivers, friends, and society. However, some of the older persons believed that in one way, requiring care gave others work opportunities. Despite their loss of physical function, the older persons also expressed gratitude for being mentally healthy and not suffering from dementia.

*“… you can be grateful as long as you are cognitively intact; that is something I feel is nice.” – 85-90 years old*

*“My life has a value since it contributes to employment for health professionals (…) they (the family) seem to value me; they do, but probably, I am just an inconvenience.” – 90-95 years old*

### A controlled death

Some of the older persons thought that they could not control death. They understood death as a force that cannot be controlled. One older person expressed that no one, not physicians or anyone else, can control death, and therefore, it is nothing to spend time worrying about. Another older woman explained that she had faith that the staff had control for her.

*“No, I do not think I have control; I trust someone else to have the control.” – 90 -95 years old*

A wish to be able to control death was described by the older persons. Some feared not being able to control their own behaviour at the moment of death. None of the older persons believed they could control their own moments of death. However, they voiced a wish to be able to say goodbye to loved ones a certain amount of time in advance of their death and a wish to be prepared for death when it occurred. At the same time, the older persons reflected that knowing when they were going to die would be distressing.

There was a strong wish among the older persons to be able to make decisions about their own death, as they considered themselves to be cognitively healthy. Several of the older persons shared thoughts about assisted dying. Some did not necessarily agree with assisted dying but were against life-supporting measures. Others expressed a positive attitude towards assisted dying and felt that decisions about one’s own death are individual decisions.

*“I have spoken with an elderly person, and she said that it should not be like this in Sweden; you should be able to decide for yourself. Because I mean, if you are clear minded, you know what you are doing … (…) Yes, then you should have the right to make decisions about your own life actually.” –80-85 years old*

The older persons’ thoughts about a controlled death concerned not only the actual moment of death but also the time afterwards. Some of the older persons wanted to be able to make decisions about their own funerals. These older persons had strong opinions about being buried or cremated. They thought about not having control or knowledge about how their bodies would be transported and stored before the actual burial ceremony, which was worrisome. These thoughts led to their awareness of the need to gain more knowledge about the process. It was important to them that they be buried at the plot or site that they and their partner had chosen.

*“I do not like burial ceremonies (…) I have a vision about how I want it.” –95-100 years old*

### Living your last period of life at a nursing home

Living at a nursing home and spending their time with other residents influenced the older persons in different ways; the older persons expressed both negative and positive attitudes, which are described in the sub-category *Living together with others*. The older persons’ outlook on everyday life at the nursing home are exemplified in the sub-category *Solitude and boredom,* which portrays the nursing home as a place characterized by isolation and a lack of stimulation.

### Living together with others

Being forced to live with others at the nursing home was described as strenuous and as having a negative impact on one’s wellbeing. The older persons perceived other residents at the nursing home to be more ill and in greater need of care than they were. Some of the older persons believed that they were in better health, both physically and cognitively, than other residents at the nursing home, which resulted in them being overlooked when it came to their own care. This led to frustration with other residents at the nursing home and feelings that the other residents did not belong there.

*“Then, some of those living here are not healthy and should not live at a nursing home (…) she has yelled at me, and that upsets me. She yells at everybody; that’s the worst.” –85-90 years old*

Some of the older persons described feeling lonely at the nursing home, suggesting a lack of community, and belonging. Factors contributing to the lack of community and sense of belonging included one’s own or others’ hearing loss, dementia, feelings of listlessness and tiredness and a lack of interest in other residents. However, some of the older persons did not experience a lack of community and belonging at the nursing home. A few of the older persons described that the nursing home provided them a social context where they were able to have meals and coffee together with other residents.

*“The people who live here, including myself, you know, it is something wrong (…) Some do not hear, and that is almost the worst when you are speaking to someone who does not hear anything, it is just what, and what and what.” – 90-95 years old*

*“… we are a few, and we can sit and maybe drink coffee and talk; I think that is a fellowship that is important. There is always someone who has something positive to talk about, and it spreads over to others.” –75-80 years old*

### Confinement and boredom

Some of the older persons characterized life at the nursing home was characterized as boring and simply satisfactory. The nursing home environment also led to a feeling of being confined and not having the same freedom as before to go outside. The possibility to go out for a short period of time was viewed as very meaningful. Some of the older persons had the ability to go out on their own, while others had to rely on family members. To be able to go out and spend time with family was viewed as very important, yet some of the older persons felt they could not rely on their families to entertain them.

*“Of course, if I did not have my daughter who is taking me out for walks and shopping, then I would probably think it was even more boring, even more dull.” – 85-90 years old*

Those who could go out on their own described this as an important part of their routines that allowed them to gain contact with the “outside world” away from the nursing home. Family was viewed as an important part of avoiding boredom and maintaining a sense of wellbeing, as many of the older persons lacked a sense of belonging. Some of the older people longed to have something to keep them busy. One man described life at the nursing home as a difficult adjustment from living a very active life to now experiencing a feeling of being confined inside the nursing home.

*“It is the lack of labour, that is something I miss. I do not know what I should say because I have always been active, and when you come down here, it is like a prison.” – 95-100 years old*

## Discussion

The aim of the study was to explore thoughts about death, dying and experiences of care in end-of-life among older persons living in nursing homes. The results emphasize that dying caused worry among older persons. Their worries were especially related to a fear of pain and a long, protracted death. The finding on the fear of dying among older persons is supported by the findings of several other studies [9, 19, 20]. The results in this study also highlight wishes among older persons at the end stage of life, i.e., dying quickly without pain or suffering quick was expressed as the ideal way of dying. The results indicate that older persons were not afraid of death, which is supported by earlier studies [6, 7, 11, 19]. In the present study, death was considered a natural and inevitable part of life. Österlind et al. [7] suggested that older persons’ lack of concerns regarding death could be explained by their view of death as a natural end to a long life.

The results in the present study showed that older persons, with a few exceptions, worried about dying but not about death. Rahm Hallberg [8] found in her literature overview that older persons tended to plan their own death, for example, their funeral arrangements. However, preparing for dying seemed to be more uncommon, even though dying was seemingly what the older persons were most worried about. A potential explanation is that older persons avoid preparation or planning because it may cause anxiety and worries. Rahm Hallberg’s [8] results are in line with the results of the present study, which show that some of the older persons had made funeral arrangements, while dying was not something the older persons were prepared for.

The purpose of this study was not to explore older persons’ opportunities to discuss death and death-related questions at the nursing home. Nevertheless, the results indicate that older persons have fears and worries about dying. These fears and worries need to be adequately addressed by caregivers. A possible explanation for why older persons worry about dying is that they are not often given the opportunity to discuss such questions [12]. It has been found that caregivers rarely open up for a discussion about death and dying [10, 12], despite the idea that older persons have a need for conversations about this topic [11, 15, 21]. Caregivers’ unwillingness to discuss death and dying has been suggested being caused by a lack of knowledge and/or confidence among caregivers [10, 11]. Thus, there exists a need to offer caregivers at nursing homes suitable support and education about how they should discuss death- and dying-related questions with older persons. The importance of giving older persons the opportunity to discuss these questions cannot be stressed enough – these conversations offer an opportunity to collect knowledge about the older person’s individual preferences in the end stage of life, and upholding these preferences should be considered an essential part of adequate end-of-life care.

The results revealed that life at the nursing home, to some extent, was characterized by negative experiences. The older persons shared stories related to the difficulties of living at a place with other residents who demanded more care than they did. The results suggest that the co-residence of mentally healthy older persons with older persons suffering from cognitive failure in the same nursing home has a negative impact on mentally healthy older persons. The older persons participating in this study were mentally healthy and expressed difficulties finding a social context at the nursing home. Previous research comparing cognitively healthy residents at nursing homes with residents suffering from dementia revealed that the former group experienced social difficulties, resulting in feelings of frustration and loneliness [26, 27]. A systematic overview by Bradshaw et al. [26] showed that mentally healthy residents avoided contact with residents with cognitive failure, which resulted in loneliness and social isolation. However, the same overview also indicated that older persons who managed to find a social context at the nursing home felt a sense of belonging that had a positive impact on their wellbeing.

Social isolation is common among older persons [28, 29], and loneliness and isolation can be connected to symptoms of depression among older persons [30]. Hence, social isolation can have a negative impact on older persons’ wellbeing [29, 30], which emphasizes the significance of optimizing older persons’ opportunities to find a social context at the nursing home. Riis Iden et al. [27] proposed dividing older persons into different wards/homes based on their mental health to improve the social aspects and quality of life of older persons in nursing homes.

### Methodological considerations

In qualitative research, trustworthiness including the concepts credibility, dependability, confirmability, and transferability, needs to be considered [25, 31] and will guide the methodological considerations of this study. One way to increase credibility is to collect data on a variety of experiences from participants to highlight several different perspectives of the phenomenon studied. This study included 20 women and 16 men aged 67 to 102 living at various nursing homes, which contributed to variation in age, gender, marital status, and nursing home type. A limitation of this study is that only older persons without cognitively impairment and who could participate during a one-hour interview were included, which might have contributed to a biased view of the final stage of life in nursing homes. Thus, it is important to highlight that the use of this sample might have prevented the results from reflecting the thoughts and experiences of the frailest, oldest persons. The data collection was conducted through structured interviews, which encouraged the older persons to respond mainly in short statements, which can be considered a limitation. It is possible that semi-structured interviews with open-ended questions would encourage more detailed and comprehensive responses. However, the older people chose to talk spontaneously about their thoughts about death and dying and their experiences of end-of-life care. It seems that the older persons had a need to talk about these issues. Thus, it can be considered that the responses, based on spontaneous thoughts that the older persons decided to share, are a strength in terms of the study credibility. To further strengthen the credibility of the study and to facilitate the reader’s assessment of the reliability of the results, quotes in each category and sub-category were presented, as well as examples of how the analysis process was performed are presented reported in Table 2. To strengthen the dependability of this study, all older persons responded to the same questionnaires, all interviews were conducted by the same experienced interviewers, and the data collection period was relatively short (9 months). The participation of both authors in the analysis process reduced the risk of misinterpretation and ensured rigour. The authors independently analysed the data but had continuous discussions during the analysis process, which strengthened the confirmability of the study. The decision if the results are transferable to other contexts has been facilitated through the provision of a clear description of the selection of participants and the context of the study for the reader. This study included cognitively healthy older persons aged 65 or older from both larger and smaller nursing homes located in both urban and rural areas. Thus, the results suggest being applicable to other older persons living in nursing homes, provided that they are cognitively healthy. However, the results are unlikely to be transferable to older persons suffering from cognitive impairment or who are not residents of nursing homes.

## Conclusions

This study indicates a need for older persons to talk about death, dying and end-of-life issues. Giving older persons the opportunity to talk about what worries them and what they perceive to be meaningful and enabling them to do so could improve their quality of life. Even though the life expectancy is not long in older persons living in nursing homes, there is a possibility that the need for care and what is perceived as meaningful change over time. Therefore, a continuous dialogue about desires and concerns via conversations with staff is necessary to improve the final stage in life among older persons. Thus, the staff in nursing homes need to be trained and supported in talking about the sensitive subjects of dying and death.

Furthermore, this study highlighted that the co-residence of cognitively healthy persons and persons with dementia in the same ward adversely affected cognitively healthy persons. This situation resulted in insufficient time to both handle the care needs of persons with dementia and have conversations that cognitive healthy persons desire. The results revealed that the needs of the cognitively healthy persons had to be deprioritized, and there was no time for them to talk about their thoughts about existence that could have given them a better quality of life. The sense of community among the older persons that might have been expected at a nursing home was lost when cognitively impaired residents were not only difficult to communicate with but were even perceived as disturbing.

## Data Availability

The datasets used and/or analysed during the current study are available from the corresponding author on reasonable request.

## Abbreviations

KUPA project: Knowledge-based Palliative Care project
WHO: World Health Organization

## References

[1] Global elderly care in crisis. Lancet, 2014, 383:927. doi: 10.1016/S0140-6736(14)60463-3. 10.1016/S0140-6736(14)60463-3 24629279

[2] Davies E, Higginson IJ (2004). Better palliative care for older people.

[3] Pivodic L, Pardon K, Morin L (2016). Place of death in the population dying from diseases indicative of palliative care need: a cross-national population-level study in 14 countries. J Epidemiol Community Health.

[4] National Board of Health and Welfare (2019). Death cause statistics 2018.

[5] Håkanson C, Öhlén J, Morin L, Cohen J (2015). A population-level study of place of death and associated factors in Sweden. Scand J Public Health.

[6] Ternestedt B, Franklin LL (2006). Ways of relating to death: views of older people resident in nursing homes. Int J Palliat Nurs.

[7] Österlind J, Ternestedt BM, Hansebo G, Hellström I. Feeling lonely in an unfamiliar place: Older people’s experiences of life close to death in a nursing home. Int J Older People Nurs. 2017;12(1). 10.1111/opn.12129. 10.1111/opn.12129 27624362

[8] Rahm HI (2004). Death and dying from old people’s point of view: a literature review. Aging Clin Exp Res.

[9] Rahm Hallberg I (2006). Palliative care as a framework for older people’s long-term care. Int J Palliat Nurs.

[10] Beck I, Törnquist A, Broström L, Edberg AK (2012). Having to focus on doing rather than being: nursing assistants’ experience of palliative care in municipal residential care settings. Int J Nurs Stud.

[11] Towsley GL, Hirschman KB, Madden C (2015). Conversations about end of life: perspectives of nursing home residents, family and staff. J Palliat Med.

[12] Alftberg Å, Ahlström G, Nilsen P, Behm L, Sandgren A, Benzein E, et al. Conversations about death and dying with older people: an ethnographic study in nursing homes. Healthcare (Basel). 2018;6(2). 10.3390/healthcare6020063. 10.3390/healthcare6020063 PMC6023469 29899220

[13] Sweden’s municipalities and county councils (2020). Fakta om äldreomsorgen i ljuset av coronaepidemin. (Facts about care for the elderly in light of the corona epidemic).

[14] Schon P, Lagergren M, Karleholt I (2016). Rapid decrease in length of stay in institutional care for older people in Sweden between 2006 and 2012: results from a population-based study. Health Soc Care Community.

[15] Smedbäck J, Öhlén J, Årestedt K, Alvariza A, Fürst CJ, Håkanson C (2017). Palliative care during the final week of life of older people in nursing homes: a register-based study. Palliat Support Care..

[16] Froggatt K, Sheila P, Morbey H, Edwards M, Finne-Soveri H, Gambassi G (2017). Palliative care development in European care homes and nursing homes: application of a typology of implementation. J Am Med Dir Assoc.

[17] Goddard C, Stewart F, Thompson G, Hall S (2013). Providing end-of-life care in care homes for older people: a qualitative study on the views of care home staff and community nurses. J Appl Gerontol.

[18] Åvik Persson H, Sandgren A, Fürst CJ, Ahlström G, Behm L. Early and late signs that precede dying among older persons in nursing homes: the multidisciplinary team’s perspective. BMC Geriatr. 2018;18(1). 10.1186/s12877-018-0825-0. 10.1186/s12877-018-0825-0 PMC6000966 29898674

[19] Fleming J, Farquhar M, Brayne C, Barclay S. Death and the oldest old: attitudes and preferences for end-of-life care - qualitative research within a population-based cohort study. PLoS One. 2016;11(4). 10.1371/journal.pone.0150686. 10.1371/journal.pone.0150686 PMC4821585 27045734

[20] Lloyd-Williams M, Kennedy V, Sixsmith A, Sixsmith J (2007). The end of life: a qualitative study of the perceptions of people over the age of 80 on issues surrounding death and dying. J Pain Symptom Manag.

[21] Seiger Cronfalk B, Ternestedt BM, Franklin Larsson LL, Henriksen E, Norberg A, Österlind J (2015). Utilization of palliative care principles in nursing home care: educational interventions. Palliat Support Care.

[22] Hall S, Petkova H, Tsouros AD, Costantini M, Higginson IJ (2004). Palliative care for older people: better practices.

[23] Ahlström G, Nilsen P, Benzein E, Behm L, Wallerstedt B, Persson M, Sandgren A. Implementation of knowledge-based palliative care in nursing homes and pre-post post evaluation by cross-over design: a study protocol. BMC Palliat Care. 2018;17(52). 10.1186/s12904-018-0308-2. 10.1186/s12904-018-0308-2 PMC5863832 29566688

[24] National Board of Health and Welfare (2020). Lägesrapport 2020 Vård och omsorg om äldre. (Progress report 2020 Care for the elderly).

[25] Graneheim UH, Lundman B (2004). Qualitative content analysis in nursing research: concepts procedures and measures to achieve trustworthiness. Nurse Educ Today.

[26] Bradshaw SA, Playford ED, Riazi A (2012). Living well in care homes: a systematic review of qualitative studies. Age Ageing.

[27] Riis Iden KR, Ruths S, Hjørleifsson S. Residents’ perceptions of their own sadness: a qualitative study in Norweigan nursing homes. BMC Geriatr. 2015;15(21). 10.1186/s12877-015-0019-y. 10.1186/s12877-015-0019-y PMC4356108 25888453

[28] Findlay C, Lloyd A, Finucane AM (2017). Experience of emotion in frail older people towards the end of life: a secondary data analysis. Br J Community Nurs.

[29] Sjöberg M, Beck I, Rasmussen BH, Edberg AK (2018). Being disconnected from life: meanings of existential loneliness as narrated by frail older people. Aging Ment Health.

[30] Vaughn L, Corbin AL, Goveas JS (2015). Depression and frailty in later life: a systematic review. Clin Interv Aging.

[31] Polit DF, Beck CT (2014). Essentials of nursing research: appraising evidence for nursing practice.

[32] World Medical Association (2013). WMA declaration of Helsinki – ethical principles for medical research involving human subjects.

[33] SFS 2003:460 (2003). Lag om etikprövning av forskning som avser människor. (Act concerning the ethical review of research involving humans).

[34] European Data Protection Board (2018). The general data protection regulation GDPR.

